# Size of the protein-protein energy funnel in crowded environment

**DOI:** 10.3389/fmolb.2022.1031225

**Published:** 2022-11-08

**Authors:** Nathan W. Jenkins, Petras J. Kundrotas, Ilya A. Vakser

**Affiliations:** ^1^ Computational Biology Program, The University of Kansas, Lawrence, KS, United States; ^2^ Department of Molecular Biosciences, The University of Kansas, Lawrence, KS, United States

**Keywords:** protein interactions, protein docking, structural modeling, cytosol, energy landscape

## Abstract

Association of proteins to a significant extent is determined by their geometric complementarity. Large-scale recognition factors, which directly relate to the funnel-like intermolecular energy landscape, provide important insights into the basic rules of protein recognition. Previously, we showed that simple energy functions and coarse-grained models reveal major characteristics of the energy landscape. As new computational approaches increasingly address structural modeling of a whole cell at the molecular level, it becomes important to account for the crowded environment inside the cell. The crowded environment drastically changes protein recognition properties, and thus significantly alters the underlying energy landscape. In this study, we addressed the effect of crowding on the protein binding funnel, focusing on the size of the funnel. As crowders occupy the funnel volume, they make it less accessible to the ligands. Thus, the funnel size, which can be defined by ligand occupancy, is generally reduced with the increase of the crowders concentration. This study quantifies this reduction for different concentration of crowders and correlates this dependence with the structural details of the interacting proteins. The results provide a better understanding of the rules of protein association in the crowded environment.

## Introduction

The geometric complementarity of interacting proteins is a key predictor of the binding modes (Vakser et al., 1999). While the local structural elements are responsible for the final lock of the proteins when their binding sites are in close proximity, there are structural factors that contribute to bringing the binding sites to such proximity. An important insight into the basic rules of protein recognition is provided by the studies of large-scale recognition factors in the absence of atom-size structural features (Vakser et al., 1999; Zhang et al., 2009), backbone complementarity in protein recognition (Vakser, 1996b), and binding-related anisotropy of protein shape (Nicola and Vakser, 2007; Vacha and Frenkel, 2011).

Large-scale structural recognition factors directly relate to the funnel-like intermolecular energy landscape (Vakser et al., 1999; Tovchigrechko and Vakser, 2001). The concept of the funnel-like energy landscapes had a profound impact on understanding of protein folding (Dill, 1999). The kinetics of the amino-acid chain folding into a unique 3D structure cannot be explained using flat energy landscapes, where minima are located on the energy surface that does not favor the native structure (so called “golf-course” landscapes). The general slope of the energy landscape towards the native structure (“the funnel”) explains the kinetics of protein folding. The basic physicochemical and structural principles of protein binding are similar, if not identical, to those of the protein folding. Thus, the funnel concept can be naturally extended to intermolecular energy (Tsai et al., 1999; Tovchigrechko and Vakser, 2001; Wolynes, 2005). As in protein folding, this concept is necessary to explain the kinetics data on protein association.

In our earlier studies, we showed that simple energy functions and coarse-grained (low-resolution) models reveal major landscape characteristics (Vakser, 1996a; Vakser et al., 1999; Tovchigrechko and Vakser, 2001; Hunjan et al., 2008; Ruvinsky and Vakser, 2008). A simplified representation of the landscape was used for a systematic study of its large-scale characteristics in a non-redundant dataset of protein complexes. The focus of the study was on the basic features of the low-resolution energy basins, such as their size, estimated by the occupancy of the low-energy clusters of docking matches (O’Toole and Vakser, 2008). The results showed that, in general, the number of such basins is small (≤10), these basins are well formed, correlated with the binding modes (the largest basins typically correspond to the experimentally determined structures of the protein assembly), and the pattern of the basins distribution, as well as the basin ruggedness, depend on the type of the complex (e.g., heterodimers typically have less rugged basins than the homodimers). The size of the intermolecular energy funnel was also estimated by generating grid-based matches and minimizing them off the grid. The minimization produced a distribution of distances, determined by a variety of metrics, between the grid-based and the off-the-grid minimized docking poses, which was used to determine the size of the binding funnel (Hunjan et al., 2008).

New computational methodologies increasingly address modeling of a whole cell at the molecular level. The whole cell modeling has many goals, such as prediction of phenotype from genotype, filling the gaps in our knowledge of cellular processes, and our ability to modulate them (Im et al., 2016; Feig and Sugita, 2019; Vakser and Deeds, 2019; Vakser et al., 2022). Many essential biochemical processes occur in the cytosolic cell compartment and various macromolecules occupy ∼1/3 of the cytoplasmic volume (Zimmerman and Trach, 1991). Thus, a key aspect of cell modeling is accounting for a crowded environment in the cytoplasm. Because of the complexity of the crowded cellular environment, most modeling approaches to crowding are based on simplified representations of the biological macromolecules, crowders, and the solvent environment (Feig et al., 2017). Biomolecular interactions in the crowded environment modulate macromolecular structure and dynamics (Yu et al., 2016). Crowding affects protein folding (Qin and Zhou, 2017), and transient protein interactions slow down protein diffusion (Nawrocki et al., 2017; Vakser et al., 2022).

Crowding environment inside the cell drastically changes protein recognition properties (Vakser and Deeds, 2019), thus significantly altering the underlying energy landscape. Adequate characterization of this landscape is essential for the modeling of protein interactions in the cellular environment. In this study we focus on the effect of crowding on the binding funnel, and specifically, on its size. Crowders occupy the funnel volume, thus making it less accessible to the cognate ligands. Since the funnel size can be defined by that occupancy (O’Toole and Vakser, 2008), it is reduced with the increase of the volume fraction of crowders in the system. In the current study, we quantify this reduction as a function of the volume fraction occupied by the crowders for several protein systems.

## Materials and methods

### Paradigm

**FIGURE 1:**
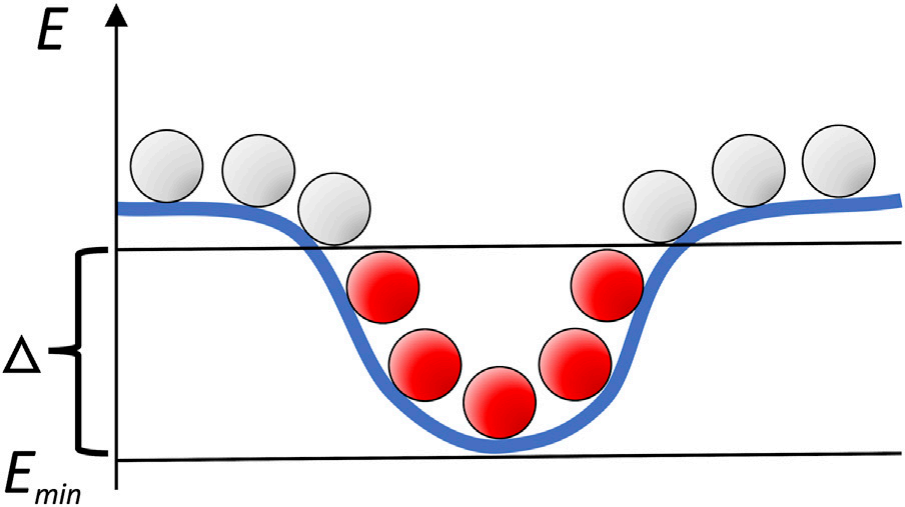
Schematic mapping of the intermolecular energy landscape by systematic sampling. Clusters of low-energy docking poses (in red) correspond to the energy basins.

Following our earlier studies of the intermolecular energy landscapes in dilute environment (Tovchigrechko and Vakser, 2001; Hunjan et al., 2008; O'Toole and Vakser, 2008; Ruvinsky and Vakser, 2008), we characterized the landscapes by distribution of the low-energy minima obtained by the Fast Fourier Transformation (FFT) docking (Katchalski-Katzir et al., 1992; Vakser, 1995). Such docking, implemented in our GRAMM procedure, systematically samples the intermolecular energy landscape on a grid (Vakser, 1996a). Thus, clusters of low-energy docking poses map the energy basins (Figure 1).

Analysis of such clusters previously allowed us to detect the intermolecular energy funnel in protein-protein complexes in isolation (dilute environment), determined to be the general characteristic of protein-protein association. The analysis was performed by comparison of the docking clusters with a random distribution of docking poses. Establishing the binding funnel’s statistical significance, however, proved to be challenging enough even in the dilute case because of the inherent difficulty of representing a nonrandom process, such as docking, by a random model (Tovchigrechko and Vakser, 2001). The crowders make such analysis far more complicated since complexes have different numbers of docking poses without clashes, thus not allowing a straightforward comparative assessment of the funnel statistical significance (Tovchigrechko and Vakser, 2001).

Thus, in this study we focused on the effective *size* of the funnel, as a function of the crowders concentration. For each complex (see Datasets), the ligand (the smaller protein in a pair) docking poses were generated by GRAMM (see Generation of the Protein Environment). We kept $N_{tot}=1000$ lowest-energy poses for further analysis. For each system of crowders (see Datasets) and for each crowder concentration (determined as the ratio of volume occupied by the crowders to the total volume of the system, hereafter referred to as crowder volume fraction), we generated 100 random different distributions (replicas) of the crowders without clashes between the crowders and those of the crowders with the receptor (the larger protein in a complex). The crowders were considered as rigid bodies, so the effects of soft interactions were ignored. For each complex *i* and replica *j*, we counted the total number of the docking poses without clashes with the crowders, $N_{tot}^{nc}\left(i,j\right)$ and the number of them in the binding site $N_{bs}^{nc}\left(i,j\right)$ (see Running Protocols). Then, for each complex *i*, we calculated the ratio (hereafter referred to as the binding site ratio)

$$\eta \left(i\right)=\frac{\underset{j}{\sum }N_{bs}^{nc}\left(i,j\right)}{\underset{j}{\sum }N_{tot}^{nc}\left(i,j\right)},$$ (1)

which was further averaged over the complexes in a dataset. In this approach, larger η correspond to the larger funnels.

Whereas in this study, we utilized the experimentally determined locations of the binding sites from the PDB, similar analysis can be performed in the absence of that knowledge. In such case, one should consider the largest cluster of the lowest-energy docking poses as such clusters usually are formed at the binding site (Hunjan et al., 2008; O'Toole and Vakser, 2008).

### Datasets

The docking data was generated using protein-protein complexes from the Dockground Docking Benchmark 4 (Kundrotas et al., 2018), chosen for convenience as a nonredundant set containing an assortment of biological functions. A subset of 141 complexes (Supplementary Table S1) was extracted based on the interface area ≥1,000 Å^2^ as determined by FreeSASA (Mitternacht, 2016), to avoid complexes with low shape complementarity previously shown to have poor discrimination of the binding site (Vakser et al., 1999; Tovchigrechko and Vakser, 2001). Of these complexes, 89 had cytosol or cytoplasm gene ontology (GO) subcellular localization term in the UniProt (Consortium, 2019; Consortium, 2021). These complexes were assigned to the cytosolic subset. The protein set was further divided according to their PDB functional classification (enzymes, signaling, binding, structural and other).

As crowders, we utilized: 1) Bovine Serum Albumin (BSA, PDB code 3v03) used in studies of crowding in cellular environment (Van Den Berg et al., 1999); 2) hen egg-white Lysozyme (HEWL, PDB code 2yvb), a small protein frequently used in experimental studies (Liu et al., 2011); and 3) the mixture of 10 glycolytic proteins used as crowders in earlier studies (Li et al., 2010) (1eou, 1eqj, 1hox, 1ig8, 1zen, 2one, 2ypi, 3gpd, 3pgk, 4pfk), chosen for their cellular abundance and evolutionary conservation. Water, ions and other small molecules were removed from the corresponding PDB files.

Calculations with the first set of the crowders (Albumin) represented the case when the crowders are, on average, larger than the target proteins (Van Den Berg et al., 2000). In the second set of studies, the crowding molecule (Lysozyme) is significantly smaller than the target protein (while still retaining more protein characteristics than classical crowders such as PEG, dextran, or Ficoll, for example). The third set of crowders approximates a more realistic cytoplasm (Li et al., 2010).

### Generation of the protein environment

**FIGURE 2:**
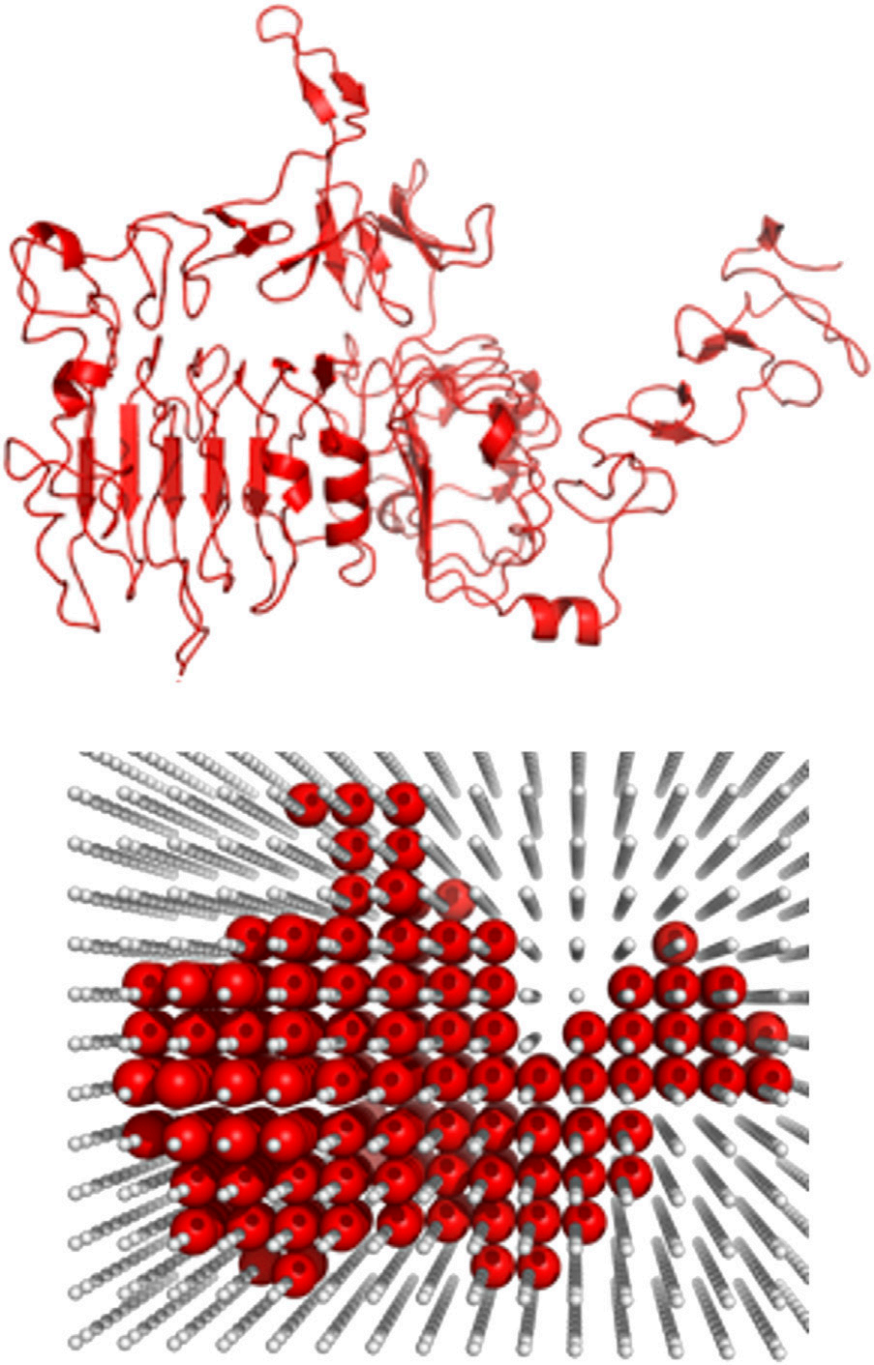
Example of the grid projection of a receptor. The receptor (1s78 chain A) is projected on a grid (grid step 6.8 Å) at the initial stage of the packing protocol.

**FIGURE 3:**
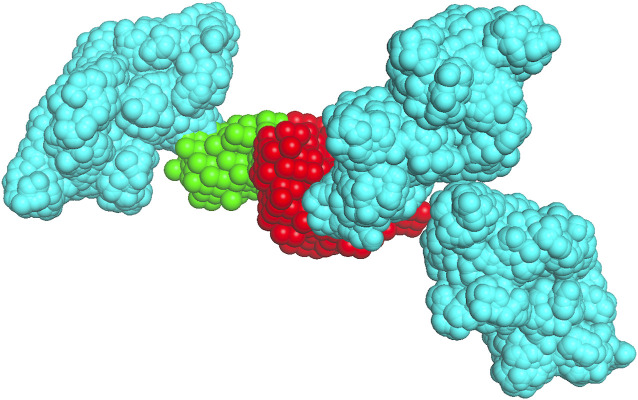
Crowder packing. The receptor (1s78 chain A) is in red, the native pose of the ligand (1s78 chains CD) is in green, and the albumin crowders at a 0.06 volume fraction (shown for clarity only within 50 Å of the receptor surface) are in cyan.

The receptor (the larger protein in the docking complex) was placed in the center of a cube with the side lengths of 870.4 Å corresponding to 128 grid steps of 6.8 Å (Vakser et al., 1999; Tovchigrechko and Vakser, 2001; O'Toole and Vakser, 2008). Figure 2 shows an example of the receptor grid projection. The size of the cube ensured that all complexes had at least two layers of crowders to minimize the edge effects. All crowders were randomly oriented and translated such that no crowder intersected any previously packed molecule (an example of crowder packing is shown in Figure 3).

Complexes were docked by GRAMM with the following parameters: grid step 6.8 Å, repulsion 6.5, and rotation interval 20° (Vakser et al., 1999; Tovchigrechko and Vakser, 2001; O'Toole and Vakser, 2008). As in our earlier studies of the intermolecular energy landscape in dilute environment, the low-resolution (coarse grain) docking parameters were selected to smooth out the local energy minima and reveal the underlying macro characteristics of the landscape (Vakser, 1996a). Docking predictions were further ranked by our AACE18 potential (Anishchenko et al., 2018) optimized for the 6.8 Å grid.

### Running protocols

**FIGURE 4:**
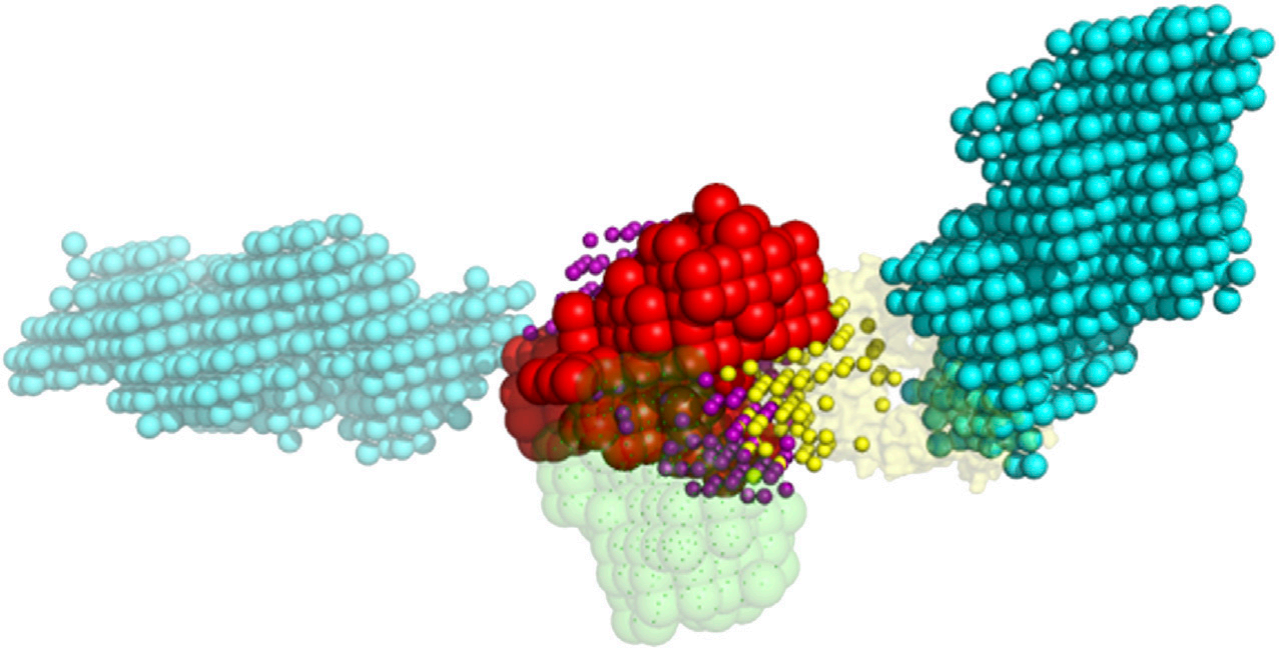
Example of the ligand poses excluded due to the clash with crowders. The red and green spheres are the grid projections of the receptor (1s78 chain A) and the native pose of the ligand (1s78 chains CD), correspondingly. The cyan spheres are the grid projection of the two BSA crowders from Figure 3. The yellow and purple spheres are the geometric centers of the docked ligand from 1,000 lowest-energy poses that do and do not clash with the crowders, respectively.

The 1,000 lowest energy ligand poses were projected on the same grid as the receptor, excluding poses that had more than two grid spaces in common with the crowders (Figure 4). The distance in 3D between the crystallographically determined geometric center of the ligand and its docked pose center was calculated. Following our earlier studies (Vakser et al., 1999) docking poses with such distance ≤10 Å were defined as those inside the binding site. The packing and analysis procedures were repeated for 100 replicas.

## Results and discussion

**FIGURE 5:**
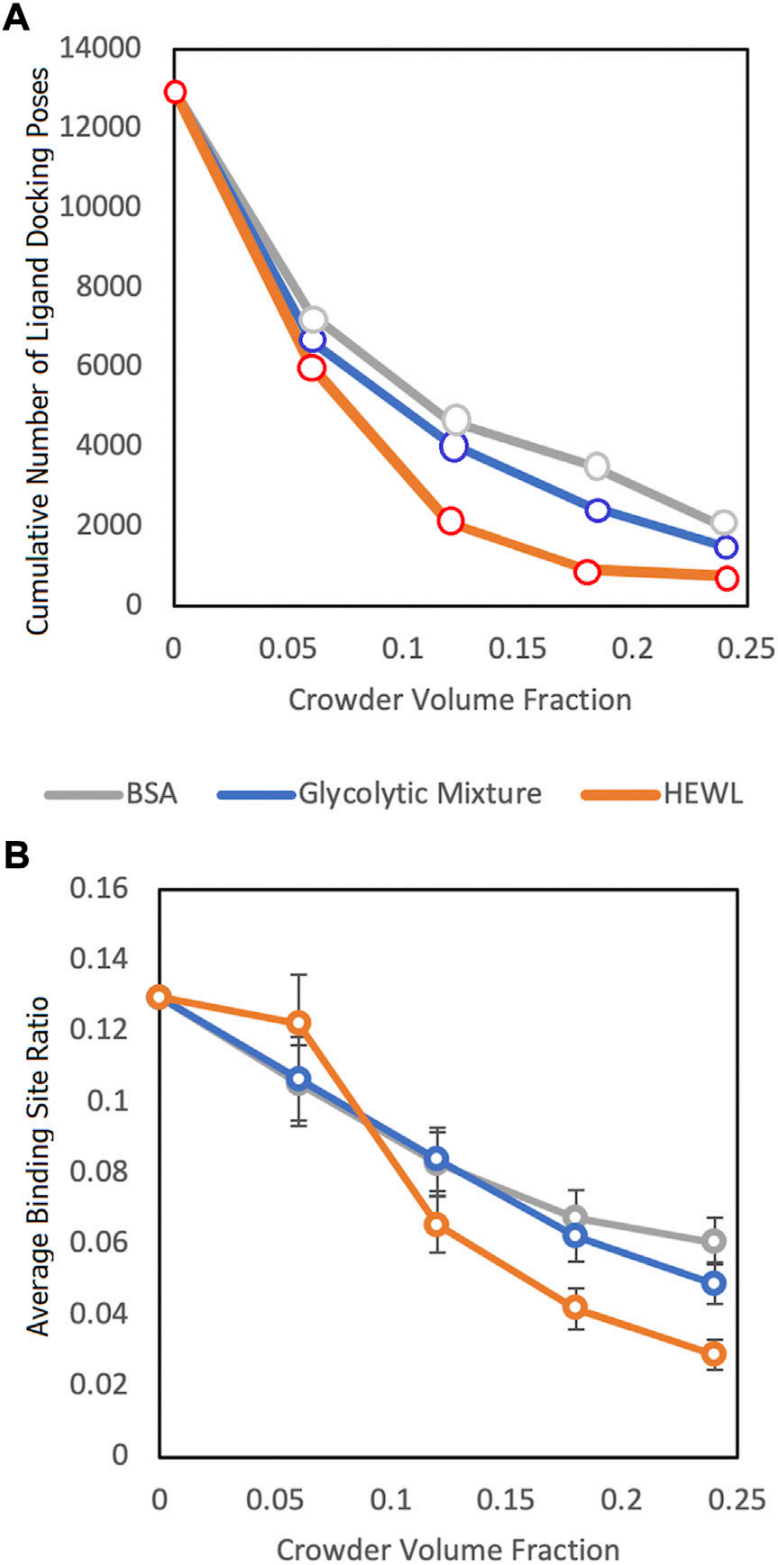
Funnel size vs. crowder volume fraction for three different crowder systems (see legend). **(A)** Cumulative number of the ligand poses in the receptor binding sites without clashes with the crowders for the entire protein-protein set and all 100 replicas. **(B)** The binding site ratio, η(i), (Eq. 1) averaged over all complexes in the entire dataset.

**FIGURE 6:**
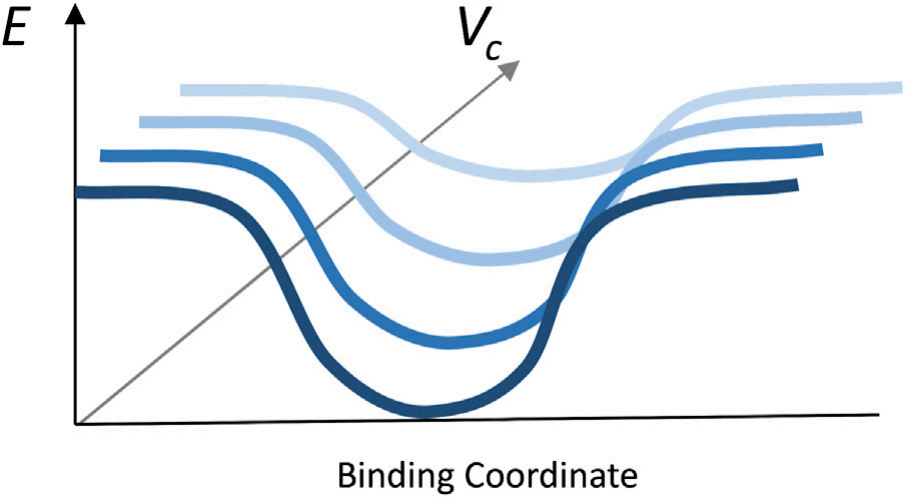
Illustration of the principle of the funnel size reduction with the increase of crowder volume fraction.

**FIGURE 7:**
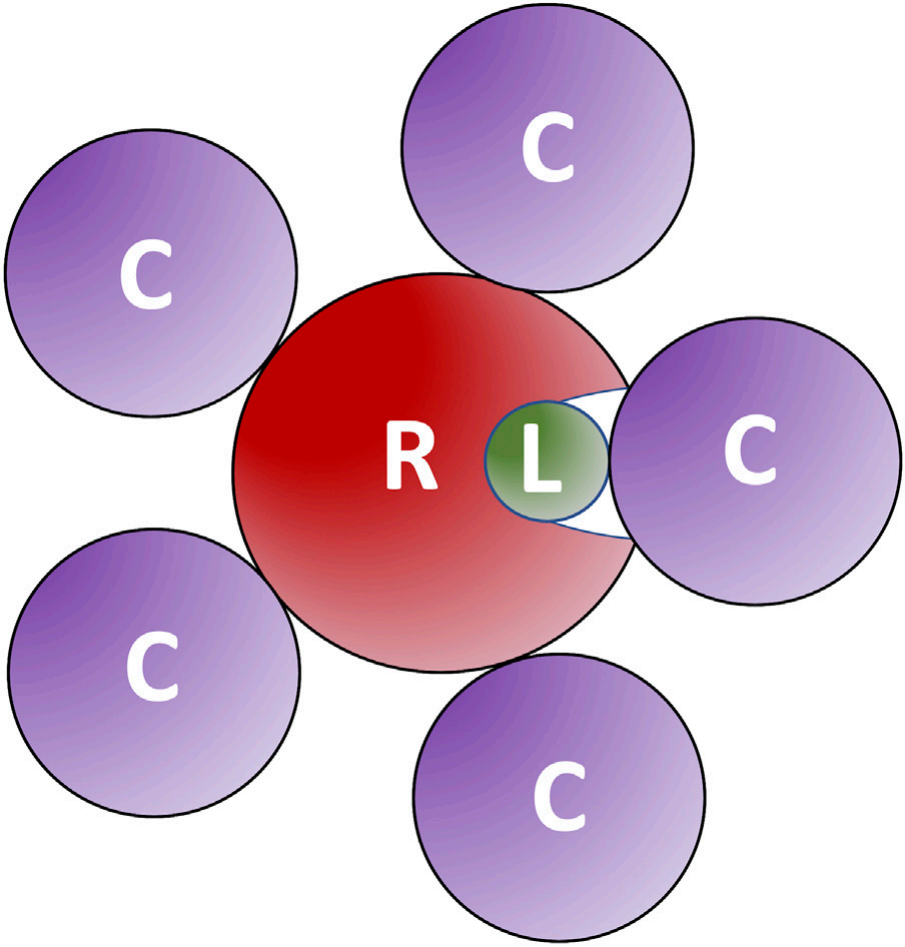
Large crowders block ligands less effectively than smaller crowders. The large crowders (C) do not fit into receptor (R) binding site to prevent ligands (L) from binding.

**FIGURE 8:**
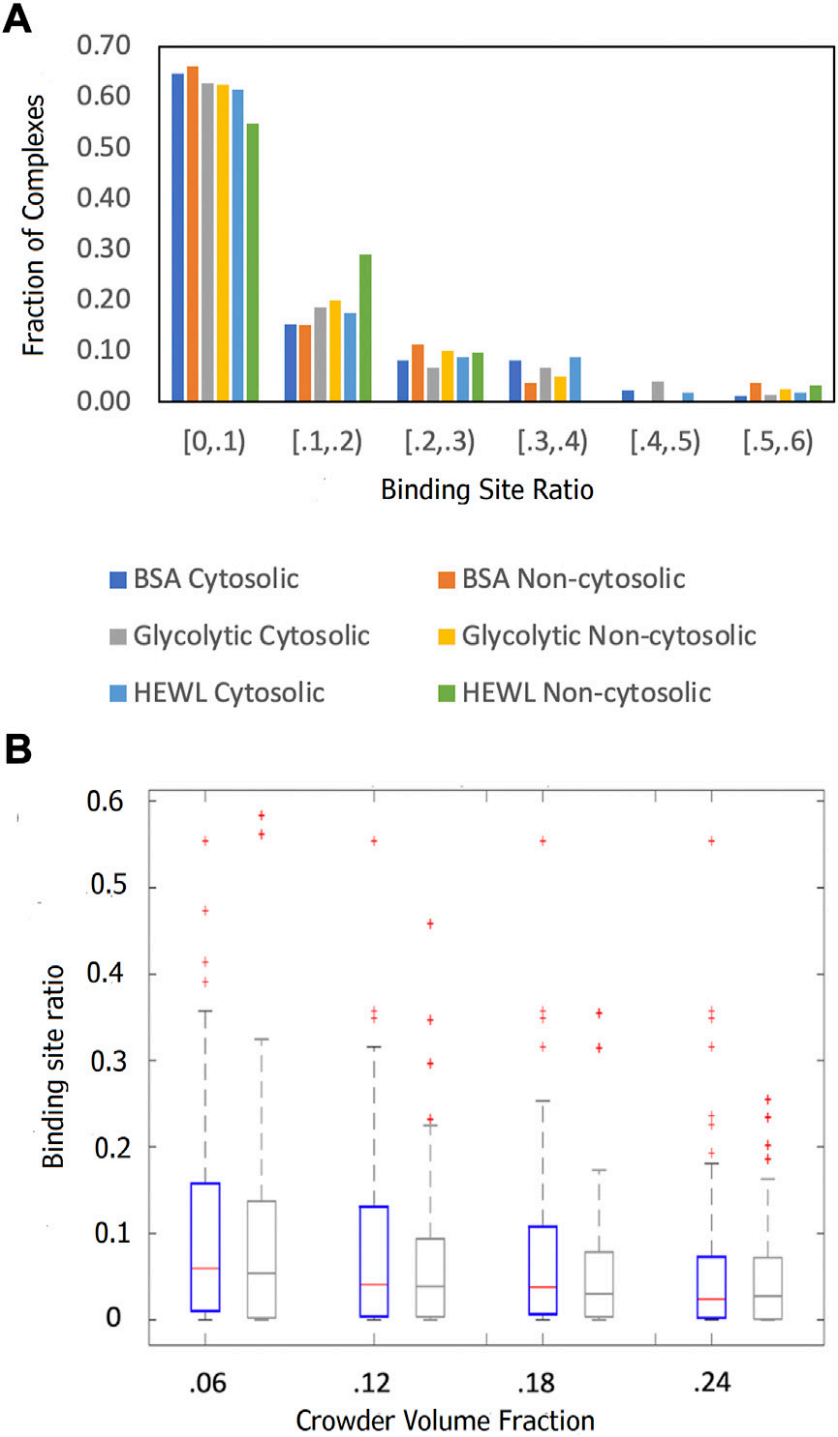
Comparison of the funnel sizes of cytosolic and non-cytosolic proteins. The data is based on 89 cytosolic and 52 non-cytosolic proteins. The funnel size for a complex i is estimated by the binding site ratio η(i) (Eq. 1). **(A)** The distributions of η(i) at 0.06 crowder volume fraction for three different crowder systems (see legend). **(B)** Box-and-whiskers representations of η(i) distributions for cytosolic (blue) and non-cytosolic (gray) proteins at different BSA crowder volume fractions.

The results (Figure 5) showed a significant reduction of the funnel size with the increase of crowder concentration for all three crowder systems (a schematic concept of this reduction is shown in Figure 6). The Albumin crowder (radius of gyration *R*_*g*_ = 36 Å) is considerably larger than the receptors in the set (average *R*_*g*_ = 25 Å). Thus, it blocks the ligands less effectively than the smaller crowders (the paradigm is shown in Figure 7). The mixture of glycolytic proteins has *R*_*g*_ ranging from 16.6 to 28.0 Å (weighted *R*_*g*_ average 23.2 Å), leading to intermediate crowding results. Crowding effect on the size of the funnel for cytosol and non-cytosol proteins, separately, shows comparable trends (Figure 8).

The binding funnel sizes were further analyzed by dividing their distribution into groups/clusters according to their numerical value (binding site ratio). The results of the 1D clustering (by MATLAB k-means analysis based on the square of the Euclidian distance) shown in Supplementary Table S2, determined that most clusters were singletons. Thus, most protein-protein complexes are unique in terms of their funnel size. Smaller crowders corresponded to fewer clusters because such crowders can pack within the binding site more effectively and thus reduce more complexes to zero binding site ligand occupancy.

**FIGURE 9:**
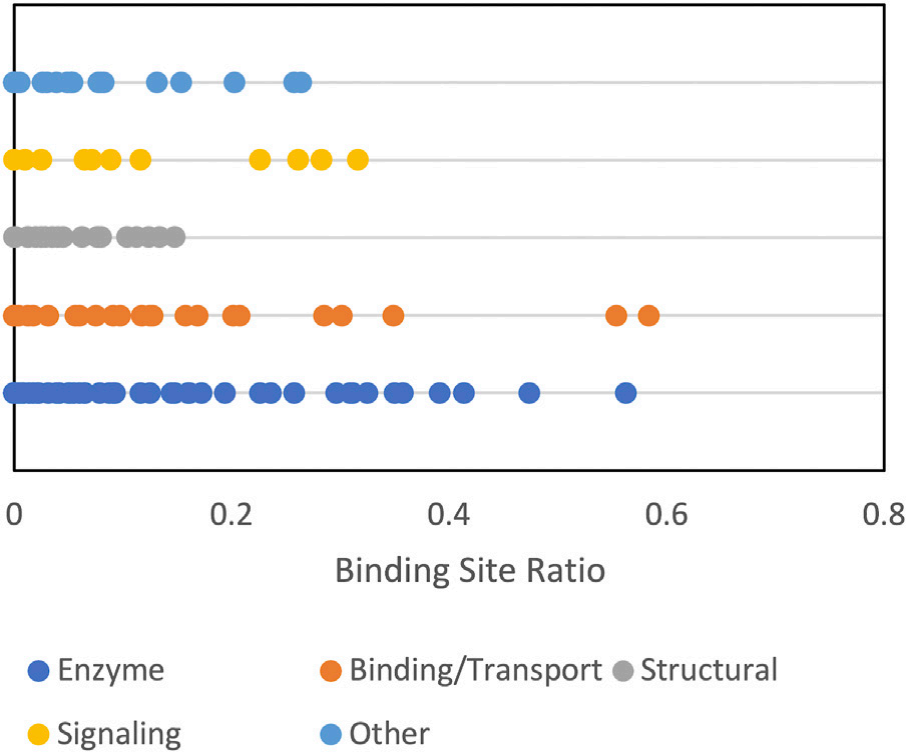
Funnel size by protein function category. The funnel size, represented by the binding site ratio (see text) is shown for albumin crowder at 0.06 volume fraction.

Supplementary Table S3 shows the five complexes with the largest funnels for different concentrations and crowder sizes. As an example, 1CLV is an enzyme binding a short peptide, which thus avoids the crowders according to our results comparing enzymes with other proteins (Figure 9). 4WLR and 1NBF are proteases binding a flexible region, so there are multiple poses of the ligand with different orientation in the binding site. However, the Albumin crowder is large enough to exclude most such ligand poses. 1Y8R is a ligase with the ligand located in a larger basin such that crowders preferentially pack near the binding site.

**FIGURE 10:**
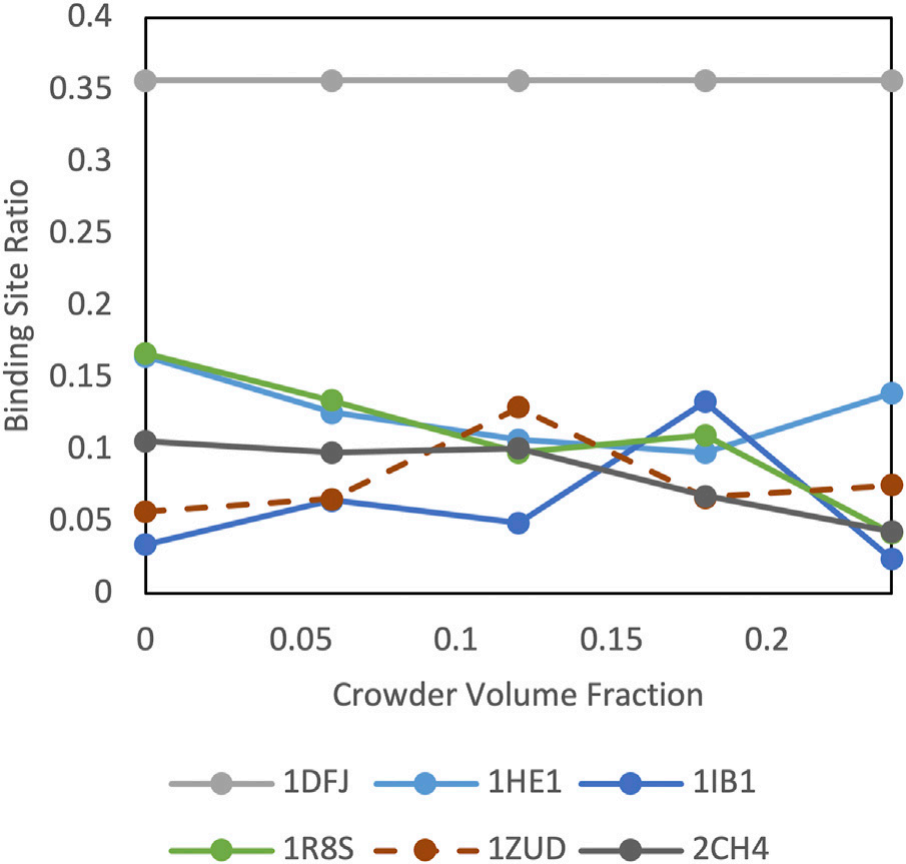
Crowding effect on special cases of cytosolic complexes.

**FIGURE 11:**
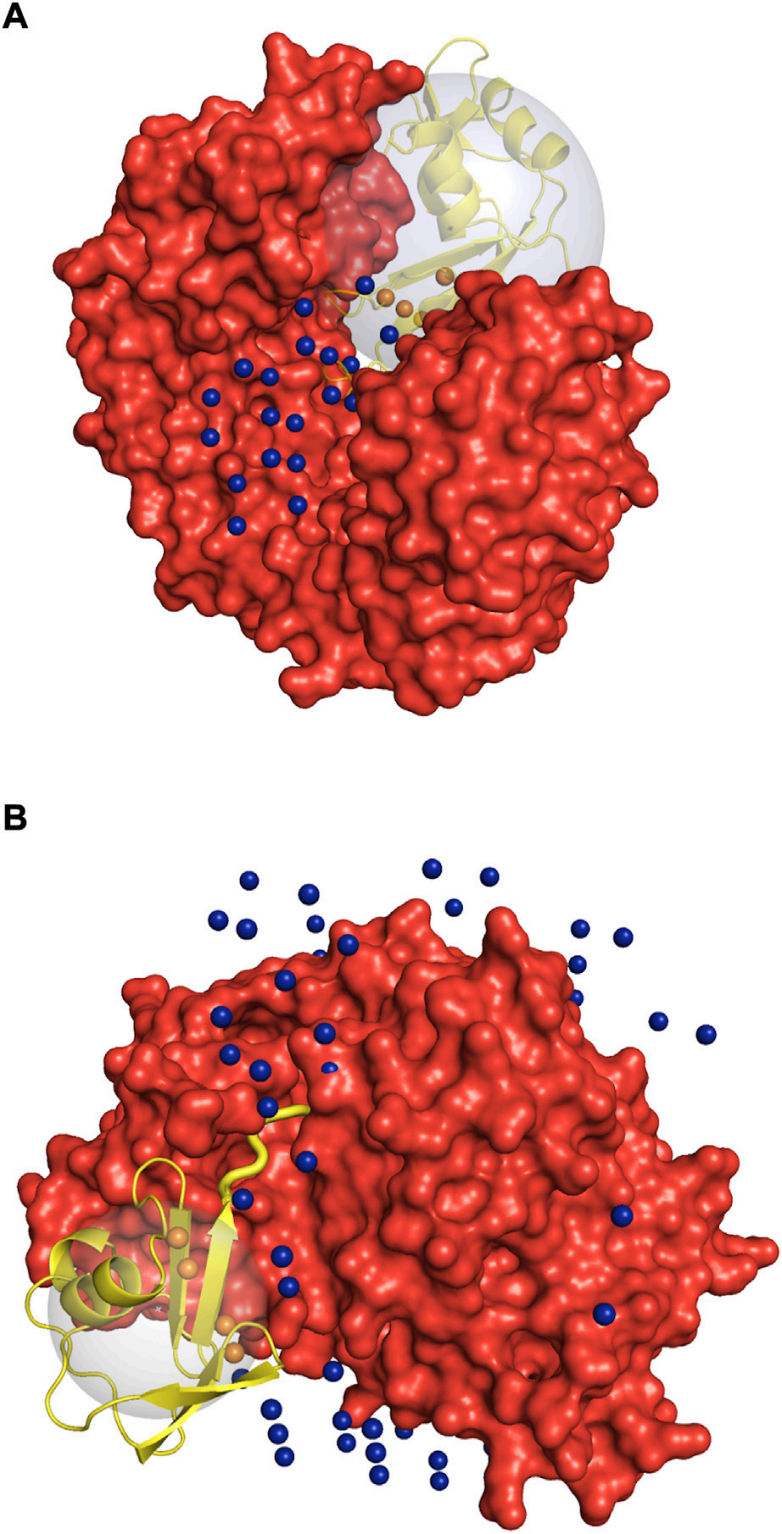
Illustration of the special cases of protein complexes. Receptor is in red, and the ligand in the native position is in yellow. The spheres are the geometric centers of the 1,000 lowest energy docked poses of the ligand inside (orange) and outside (blue) the binding site. The large gray sphere shows the space in which the ligand poses (geometric centers) are considered to be within the receptor binding site. Due to the low-resolution grid-based nature of the docking procedure used in this study, one geometric center can correspond to multiple docking poses with different rotational orientation. **(A)** 1dfj complex: the enzymatic binding site on the receptor is too small for the crowders to fit in. **(B)** 1zud complex: C-terminal of the ligand (thick yellow cartoon) binds inside a deep groove on the receptor surface. See text for details.

Protein shape characteristics determine the funnel size in the dilute environment (Hunjan et al., 2008; O'Toole and Vakser, 2008; Ruvinsky and Vakser, 2008). Similarly, they affect the dependence of the funnel size on the crowder concentration. For example, enzymes tend to have deep binding pockets (Ben-Shimon and Eisenstein, 2005), which shield ligands from the crowders (Figure 7). This prominent shape characteristic is a likely reason for the enzymes to have the largest energy funnel among protein function categories (as shown in Figure 9 for 0.06 BSA crowder concentration). Still, on average, the funnel for the enzymes gets smaller with the increase of the crowder concentration. In an extreme case, however (Figure 10, 1dfj) an enzyme had a constant funnel size at all crowder concentrations. Because of the shape of the receptor in that complex, all 1,000 lower-energy (higher shape complementarity) docking positions were located inside semicircular cavity of the receptor, which is inaccessible to the crowders (Figure 11A).

Protein complex 1zud has a sharp maximum at the medium concentration of crowders (Figure 10). In this complex, the C-terminal of the ligand binds inside a deep groove on the receptor surface (thick yellow cartoon in Figure 11B). The low resolution of the docking procedure used in this study does not capture such shape characteristic. Thus, 1,000 lowest-energy matches are located predominantly far from the receptor binding site (Figure 11B). Therefore, it is more likely that the crowders first encounter docking poses outside the binding site, thus increasing the binding site ratio *η*(*i*) for this complex. Only when the number of crowders increases, they start excluding docking poses inside the binding site, leading to the decrease of *η*(*i*).

Signaling proteins were often represented in our dataset by more than one complex per pair of proteins, pointing to alternative binding modes. Supplementary Figure S1A shows that signaling is a common GO biological process listed in the UniProt for the proteins in the set. Presence of the alternative binding modes decreased the estimated funnel size (Figure 9) because some ligand matches went to the alternative site.

Two factors contribute to the large representation of membrane localization in Supplementary Figure S1B. The cytosolic set is defined as the union of all subcellular localization terms assigned to either receptor or ligand such that the resultant set has at least one occurrence of “cytosol” or cytoplasm, which allows cytosolic and membrane localization terms to coexist since each protein may be involved in multiple biological processes. For example, if the receptor protein was annotated as “membrane” and “cytosol,” and the ligand only as “membrane,” the complex was designated as “cytosol.” Second, proteins at the cytosol-membrane interface perform several functions and consequently receive multiple membrane related annotations. The larger share of smaller funnels for non-cytosolic proteins with the Lysozyme crowder (Figure 8A) is because Lysozyme, as opposed to the larger crowders, is small enough to fit into the b-barrel receptor (Supplementary Figure S2), thus obstructing ligand binding more effectively.

In general, our results are consistent with predictions from the free-volume theory (Asakura and Oosawa, 1954) that exclusion effects increase with increasing crowder concentration and larger crowders exclude less volume than smaller crowders.

## Conclusion

Protein-protein binding modes to a significant extent are determined by the geometric complementarity of the interacting proteins. Large-scale recognition factors provide important insights into the basic rules of protein recognition. The large recognition factors directly relate to the funnel-like intermolecular energy landscape. The concept of the funnel-like energy landscapes had a profound impact on understanding of protein folding and binding. Previously, we showed that simple energy functions and coarse-grained models reveal major landscape characteristics. As new computational approaches increasingly address structural modeling of a whole cell at the molecular level, accounting for a crowded environment in the cytoplasm becomes a key modeling aspect. The crowded environment drastically changes protein recognition properties, and thus significantly alters the underlying energy landscape. In this study we focused on the effect of cellular crowding on the protein binding funnel, specifically addressing the funnel size. Crowders occupy the volume of the funnel, making it less accessible to the ligands. Thus, the funnel size, which can be defined by that occupancy, in general, is reduced with the increase of the crowders concentration. Our study quantified this size reduction for different concentration of crowders and correlated the size vs. concentration dependence with the structural details of the interacting proteins. Our plans for future research include systematic studies of crowding effect on other characteristics of the intermolecular energy landscape, such as the number of funnel-like energy basins and the landscape transformation that follow changes in the force field. The landscape characteristics will be correlated with the kinetic properties of the system (Vakser et al., 2022). The studies should provide better understanding of the rules of protein association in the crowded environment, as computational biology moves toward structure-based modeling of the cell.

## Data Availability

The original contributions presented in the study are included in the article/Supplementary Material, further inquiries can be directed to the corresponding authors.
