# Supplementary material for: Size of the protein-protein energy funnel in crowded environment
Source: Front Mol Biosci. 2022 Nov 8;9:1031225. doi: 10.3389/fmolb.2022.1031225 (PMC9679368; doi:10.3389/fmolb.2022.1031225)
Supplement: Supplementary file 1 [file DataSheet1.PDF]

Supplemental Material

Table S1. *S*tructures used in the study.

1AKJ:AB.DE[1]	1X86:A.B	3CII:ABC.GH	4FA8:ABCDHI.FG	4X6Q:B.C	1BGX:HL.T	3GE3:ABCDFG.H	4KR0:AC.B
1DE4:AB.CF	1XK4:AB.D	3CVH:ABC.HL	4FME:A.B	4XR8:AF.C	1BUI:AB.C	3L89:ABC.M	4M7L:HL.T
1DFJ:E.I	1XT9:A.B	3CW2:A.K	4HCN:A.B	4Y5O:A.B	1BVN:P.T	3LTF:AC.D	4ML7:A.B
1G4B:KLQRWX.MNOPSTUVYZab	1Y64:A.B	3DAW:A.B	4HRE:C.EFGH	4YOC:A.C	1CLV:A.I	3MJ9:A.HL	4P1B:ABCDEF.I
1GG2:A.B	1Y8R:AB.C	3F7P:A.C	4I5L:AC.B	4ZFR:A.B	1JIW:I.P	3OED:A.C	4PDC:AB.E
1GPW:AB.C	1ZUD:13.4	3FPU:A.B	4K71:A.BC	5BRR:E.I	1JTD:A.B	3PRO:A.C	4TXV:A.B
1HE1:A.C	2BCG:G.Y	3G6D:A.HL	4LNU:ABD.K	5C3I:I.JKL	1ML0:AB.D	3RNK:A.B	4WZA:ABCD.GH
1HYR:AB.C	2BKU:A.B	3K9M:A.C	4LRZ:A.FH	5CNV:AB.FG	1R5I:ABC.D	3T1Q:A.BC	4XIG:MNST.Q
1I2M:A.B	2C0L:A.B	3LWN:A.F	4MNE:BC.A	5EE4:A.CD	1TMQ:A.B	3VLB:A.B	5AUP:BI.H
1IB1:B.F	2CH4:A.W	3PNL:AC.B	4NIF:AD.B		1U0N:AD.BC	3ZN6:A.BD	5C7X:A.HL
1IS7:ABCDEFGHIJ.PQRST	2J12:ACE.B	3QLU:A.C	4NYI:Q.S		1UUG:A.B	4CU4:A.B	5CEC:A.B
1JK9:A.B	2NXN:A.B	3R9A:AC.D	4OL0:A.B		1V5I:A.B	4DI3:ABC.E	5E0K:C.BD
1JZD:AB.C	2RII:AB.X	3RRM:AB.C	4P4H:EFGHUWX.MNOP		1ZLH:A.B	4ETQ:C.HL	
1K5G:C.A	2X0B:E.F	3RVD:ABCD.I	4P69:B.CD		2A1T:ABCD.RS	4FI3:ABCD.F	
1NBF:A.D	2ZAE:A.B	3UAI:ABC.D	4PJ8:AB.CD		2BQ1:EF.IJ	4GAM:ABCFGH.D	
1NW9:A.B	2ZVN:A.BD	3VXU:ABC.DE	4QD2:ABCD.E		2GRX:A.C	4HH3:AB.C	
1R8S:A.E	3A1P:A.B	3ZNZ:A.B	4RIX:A.B		2HQS:A.H	4HQP:ABCDE.G	
1S78:A.CD	3BH6:A.B	4C0O:A.C	4RWS:A.C		2UY7:A.B	4HX3:BD.C	
1VG0:A.B	3BX7:CD.A	4CT4:A.B	4WJG:ABC.E		3AV0:A.B	4J4L:A.C	
1WQ1:G.R	3CBK:A.B	4EJX:A.BD	4WLR:AB.C		3E2L:A.C	4JO9:AC.B	

[1] Cytosolic proteins are on gray background. PDB code of the structure is separated by a column from the chain IDs. Chains corresponding to receptor and ligand are separated by a dot.

Table S2. Binding funnels clustered according to size.

	Crowder Volume Fraction														
	0			.06			.12			.18			.24		
	Albumin	Glyc.	Lysoz.	Albumin	Glyc.	Lysoz.	Albumin	Glyc.	Lysoz.	Albumin	Glyc.	Lysoz.	Albumin	Glyc.	Lysoz.
No. of Clusters	83	83	83	110	94	81	110	94	93	110	94	85	99	92	79

Table S3. Complexes with largest funnel size constant across concentrations and crowder sizes.

	Crowder Volume Fraction														
	0			.06			.12			.18			.24		
Top Complexes	Album.	Glyc.	Lysoz.	Album	Glyc	Lysoz.	Album.	Glyc	Lysoz.	Album.	Glyc.	Lysoz.	Album.	Glyc.	Lysoz.
	1CLV	1CLV	1CLV	4CU4	1CLV	1CLV	1I2M	1CLV	1CLV	1I2M	1CLV	1CLV	4CU4	1CLV	1CLV
	4WLR	4WLR	4WLR	1CLV	4WLR	1I2M	4CU4	4WLR	4WLR	1DFJ	4WLR	1XT9	2J12	4WLR	1XT9
	1NBF	1NBF	1NBF	1I2M	1I2M	4WLR	1DFJ	4ZFR	1NBF	1CLV	4ZFR	4WLR	1I2M	4ZFR	4WLR
	4ZFR	4ZFR	4ZFR	4WLR	1NBF	1NBF	4OL0	1NBF	1ML0	4OL0	1XT9	1NBF	3RVD	1NBF	1NBF
	1Y8R	1Y8R	1Y8R	4ZFR	4ZFR	1DFJ	1CLV	1XT9	1XT9	3BH6	1NBF	1ML0	1DFJ	1TMQ	4ZFR

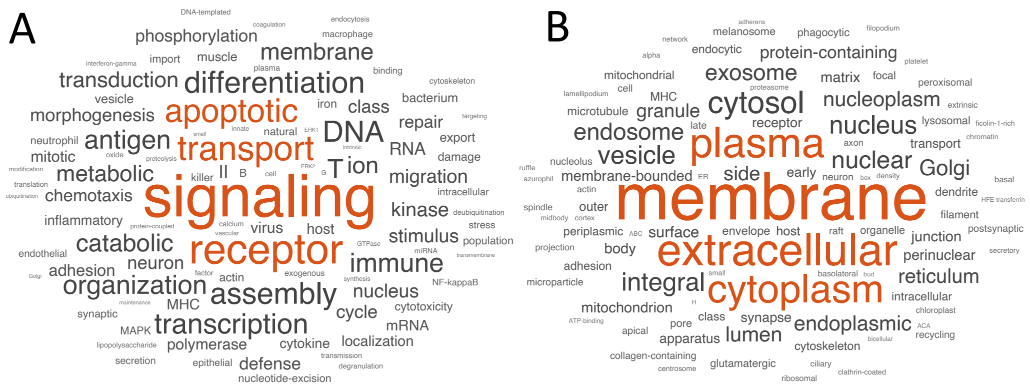

Figure S1. *Composition of the protein dataset*. The word cloud shows all terms found in the GO (A) biological process and (B) cellular component fields of the UniProt for all ligands and receptors in the data set.

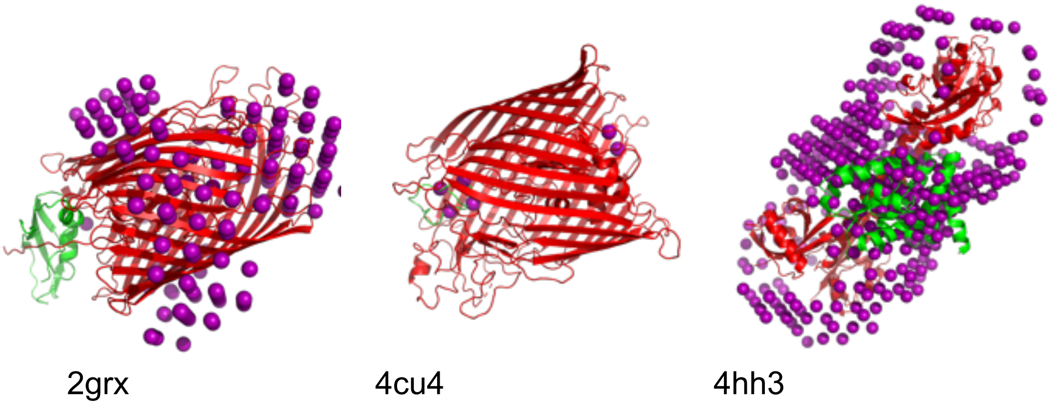

Figure S2. *Examples of docking to membrane proteins*. The receptors are in red, ligands in the native pose in green, and ligand docked poses in purple. Lysozyme crowders are small enough to exclude ligand poses in the binding site.
